# A dataset of thermal preferences for Mediterranean demersal and benthic macrofauna

**DOI:** 10.1038/s41597-024-03168-5

**Published:** 2024-03-27

**Authors:** Salvatore Valente, Francesco Colloca

**Affiliations:** 1 Department of Integrative Marine Ecology, Stazione Zoologica Anton Dohrn, Rome, Italy; 2 Department of Biology and Biotechnologies ‘Charles Darwin’, Sapienza University of Rome, Rome, Italy

**Keywords:** Climate-change ecology, Biodiversity, Animal physiology

## Abstract

Climate change is swiftly reshaping marine ecosystems, affecting different biological levels. Changes in thermal conditions profoundly influence ectotherms’ growth, behaviour, and functions, making knowledge of species’ thermal preferences (TP) crucial for understanding their responses to ongoing warming. However, obtaining this data is challenging due to limited studies, especially for deep-sea demersal and bottom-dwelling species. Here, we present the MedFaunaTP dataset, a collection of survey-based TPs for 939 Mediterranean species of fish, crustaceans, molluscs, echinoderms, cnidarians, and tunicates calculated using species abundance data obtained from the international bottom-trawl survey in the Mediterranean (MEDITS) and bottom temperature data derived from the Copernicus Monitoring Environment Marine Service. MEDITS estimates are based on species biomass indices from 27587 sampling stations, collected from 1994 to 2020, covering the northern Mediterranean Sea and spanning depths from 10 to 800 m. The MedFaunaTP dataset may serves as a valuable resource for understanding and addressing marine ecosystem ecological, conservation, and management challenges in the context of climate change and associated global warming.

## Background & Summary

Climate change is rapidly altering the structure and functioning of marine ecosystems, impacting all biological levels and interacting with human activities like fishing^1–3^. Temperature profoundly influences the fitness of organisms, especially ectotherms, as their physiological processes are largely temperature-dependent^4,5^. Since ectotherms’ physiology is governed by ambient temperature, variations in thermal conditions significantly impact their somatic growth, behavior and other vital functions^4,6^. Physiological changes can lead to shifts in mortality rates, growth patterns, reproductive behaviors, as well as alterations in the phenology and distribution of populations (e.g.^1,7,8^). Therefore, knowing species thermal preference is pivotal to understand how they are reacting to the ongoing warming. However, such information is often lacking especially for species living in remote and inaccessible ecosystems^9^ such as the deep-sea. As far as we are aware, there are two main sources that offer information regarding the thermal preferences of marine species: AquaMaps^10^ and the dataset provided by Cheung *et al*.^11^. AquaMaps has been used to infer thermal preferences of a variety of marine species using species distribution models and environmental data. However, Aquamaps species distribution models are uncertain and predictive accuracy varies^12^. Cheung’s dataset is limited to fish species and relies on sea surface temperature data, which doesn’t fully encompass the thermal preferences of bottom-dwelling species, particularly those inhabiting the deep-sea. Furthermore, none of these datasets are specific to populations in the Mediterranean Sea which, as semi-enclosed basin, exhibits substantial differences in environmental conditions compared to the nearby Atlantic Ocean.

To bridge this knowledge gap, we expanded upon the methodology proposed by Valente *et al*.^13^. We used survey-based species abundance data obtained from the international bottom-trawl survey in the Mediterranean (MEDITS^14^) in combinations with measurements of bottom temperature derived from the Copernicus Monitoring Environment Marine Service to estimate survey-based TP. The resulting MedFaunaTP dataset provides thermal preference estimates for 939 Mediterranean species, encompassing six main taxa such as fish, crustaceans, molluscs, echinoderms, cnidarians, and tunicates. The MedFaunaTP dataset represents a valuable resource for understanding and addressing the impact of global warming on the Mediterranean marine ecosystem. The Mediterranean region is indeed regarded as a climate change hot spot, warming 20% faster than the global average^15^. Its biological communities are facing big challenges, including a long history of overfishing^16^, species range shifts caused by warming temperatures^1^, and increased occurrence of non-indigenous invasive species of Indo-Pacific origin^17^.

## Methods

### MEDITS trawl survey data

**Fig. 1:**
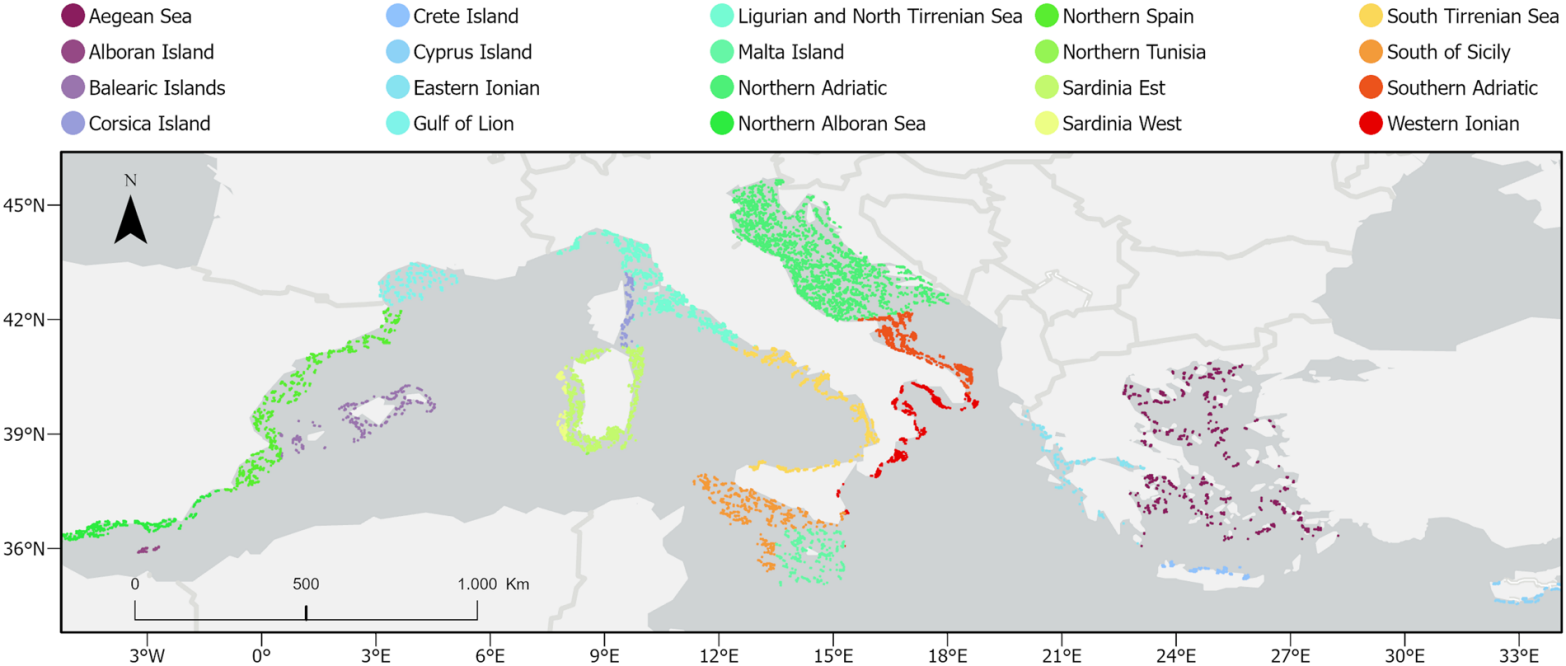
Study area and MEDITS survey sampling stations sampled between 1994 and 2020.

Species abundance data were obtained from the MEDITS bottom trawl survey^14^ which is carried out annually in May-July since 1994. The survey covers both the continental shelf (10 to 200 m depth) and the continental slope (200 to 800 m) of an area extending for roughly 543000 km^2^ (from 34.33°N to 45.67°N and 5.22°W to 34.09°E) and enclosing the coasts of 10 EU countries: Spain, France, Italy, Greece, Slovenia, Croatia, Albania, Montenegro, Malta and Cyprus. In recent years, African countries participating in the MedSea4Fish program (https://www.fao.org/gfcm/activities/fisheries/cooperation/medsea4fish), under the auspices of the General Fisheries Commission for the Mediterranean (GFCM), have conducted similar scientific bottom trawl surveys. However, these surveys begun after 2019, with Tunisia being the only participant at that time. Consequently, a significant portion of this data falls outside the time series for EU Mediterranean countries that we used in this study. Furthermore, these data are not archived into a shared database and are not governed by a clear data policy for access and dissemination. Consequently, each national dataset would need to be formally requested from each country individually, entailing a lengthy and uncertain data gathering process. Sampling procedures follow a standardized protocol: all the vessels involved in the survey are equipped with the same bottom trawl net, known as GOC-73, using cod-end mesh size of 20 mm. The net had an average vertical opening of 2 meters and a wing-span of 18 meters^14^. All trawling operations were conducted during daylight hours. To ensure consistency, both the trawl speed and tow duration were set at 3 knots and 30 minutes for stations located on the continental shelf and 60 minutes for stations on the slope. During each haul, data on sampling depth, tow duration, swept area (i.e., the trawled surface), and geographical coordinates were systematically collected. At the end of each haul, all the organisms collected were separated by species, counted, and weighted to obtain abundance (n km^−2^) and biomass (kg km^−2^) indices. The species considered during the survey included, in addition to demersal organisms (fish, cephalopods and crustacean decapoda and stomatopoda), also macro-epibenthic organisms belonging to several taxa. For the purpose of this study, we extracted the biomass indices (kg km^−2^) of species caught in a total of 27587 hauls carried out in the period 1994–2020 (Fig. 1). We excluded from the analysis the less frequent and abundant sampled taxa such as Polychaeta and Porifera, thus focusing on Teleostea, Chondrichthyes, Crustacea Decapoda and Stomatopoda, Mollusca Cephalopoda, Bivalvia e Gastropoda, Echinodermata, Cnidaria, and Tunicata.

### Bottom temperature data

We obtained monthly-mean bottom temperature (*BT*) data for the Mediterranean Sea from the Copernicus Monitoring Environment Marine Service’s Mediterranean Sea Physics Reanalysis service^18^ for the period 1994–2020. For each year, we calculated the average BT value for each quarter of the year (Q1, Q2, Q3, and Q4). Spatial resolution was defined on a regular grid at 1/24 degree (~4 km).

### The mean thermal preferences

We used biomass indices (*C*) of demersal and macro-epibenthic species and *BT* data to estimate their annual thermal preferences (*TP*) using the following equation:

1 $$T{P}_{j,yr}=\frac{{\sum }_{i}^{n}B{T}_{i}{C}_{j,i,yr}}{{\sum }_{i}^{n}{C}_{j,i,yr}}$$

where *C*_*j,i,yr*_ represents the biomass of species *j* in haul *i* collected during the year *yr*, *BT*_*i*_ is the average bottom temperature for the quarter of the year when and where the haul *i* was carried out (e.g., hauls sampled in May 2000 were associated with the average bottom temperatures of the second quarter of 2000), and *n* is the total number of hauls collected during the year *yr*. The mean thermal preference of each species is obtained by averaging the yearly thermal preference of each species.

## Data Records

The final data set consisted of 939 Mediterranean species: 369 fish, 218 molluscs, 181 crustaceans, 70 echinoderms, 46 tunicates and 55 cnidarians. Species names were retrieved from the taxonomic list of the MEDITS program (https://www.sibm.it/MEDITS%202011/revision%20TM%202017.htm). Five fields were associated with each species (Table 1).

**Table 1:** Details of the variables used in the data file “PrefTemp.csv”.

Column	Variable identity	Variable definition	Units of measurement	Storage type	Range values	Length
1	Scientificname	Species name according to the species list of MEDITS.	N/A	Character	N/A	Variable
2	Taxon	Species taxon according to MEDITS taxonomic list.	N/A	Character	N/A	Fixed
3	PrefTemp	Species TP estimated as in the Eq. (1).	°C	Numeric	12.06 to 26.61	Fixed
4	FO	Frequency of occurrence for each species.	%	Numeric	0.00 to 77.69	Variable
5	CV	Coefficient of variation	—	Numeric	0.00 to 0.35	Fixed

N/A = Not Applicable.

The MedFaunaTp dataset is stored as a csv file and available for download on Figshare data repository^19^.

Along with the MedFaunaTp dataset, we have made available a Network Common Data Form (NetCDF) file encompassing 108 raster datasets of bottom temperatures, which served as the source for extracting values at corresponding haul positions. Each variable name within the NetCDF file follows a structured format: “BT_“ followed by the year and quarter (e.g., “BT_1994_Q1” represents bottom temperature data for the first quarter of 1994). This file is named “BTrasters.nc” (file size: 159 MB).

## Technical Validation

Species names provided in the MEDITS taxonomic list were checked with the World Register of Marine Species (WoRMS^20^, accessed 2023-10-26) to take into account recent taxonomic changes. For each species collected in at least two sampling years, we computed the coefficient of variation (CV) based on annual estimates of TPs to evaluate the interannual variability of the index. Subsequently, we generated box plots to illustrate the range of variation for each investigated species. For most of the species the CV of *TP* estimates was below 20% showing that their thermal preferences were substantially stable during the study period (Fig. S1 -Supplementary Information). The CVs of each species were included into the MedFaunaTp dataset under the column “CV” (Table 1).

Furthermore, given that there are large differences in abundance among species we also included the species frequency of occurrence (n of positive hauls/total n of hauls) in the MedFaunaTp dataset, adding a column labelled “*FO*”. *FO* provides a supplementary indicator of the reliability of the *TP* estimates associated with each species, assuming that the higher the “*FO*” the more reliable the TP estimate.

### Supplementary information

Supplementary Information

## Data Availability

All the analyses were conducted within the R environment (R version 4.2.1). We utilized *raster* and *terra* packages^21,22^ to create and structure raster datasets, while *ncdf4* package^23^ was employed to handle NetCDF files. Additionally, data pre-processing and wrangling tasks were performed using the *tidyverse* package^24^. The code for downloading and generating the raster files can be found in the “bottomT.R” script. Unfortunately, we are unable to share the code pertaining to the *TP* estimates as the authors of this paper are not authorized to distribute the raw MEDITS data. Distribution and use of the MEDITs dataset is regulated by EU Regulation 2017/1004^25^.
