# Supplementary material for: A dataset of thermal preferences for Mediterranean demersal and benthic macrofauna
Source: Sci Data. 2024 Mar 27;11:314. doi: 10.1038/s41597-024-03168-5 (PMC10973485; doi:10.1038/s41597-024-03168-5)
Supplement: Supplementary file 1 — Supplementary Information [file 41597_2024_3168_MOESM1_ESM.pdf]

Supplementary Information

A dataset of thermal preferences for Mediterranean demersal and benthic macrofauna.

Salvatore Valente[1,2], Francesco Colloca[1]

1. Department of Integrative Marine Ecology, Stazione Zoologica Anton Dohrn, Rome, Italy.

2. Department of Biology and Biotechnologies 'Charles Darwin', Sapienza University of Rome, Rome, Italy.

Corresponding author(s): Salvatore Valente (salvatore.valente@uniroma1.it)

SUPPLEMENTARY CAPTION

These figures can be collectively regarded as Figure S1. We utilize box plots to illustrate the range of variation in the annual estimates of thermal preferences for species within the specified taxa. For improved clarity, the box plots are color-coded, with green denoting a coefficient of variation (CV) less than or equal to 0.2, while red represents CV values exceeding 0.2. Grey box plots correspond to species collected only in one sampling year, for which CV calculations were not feasible.

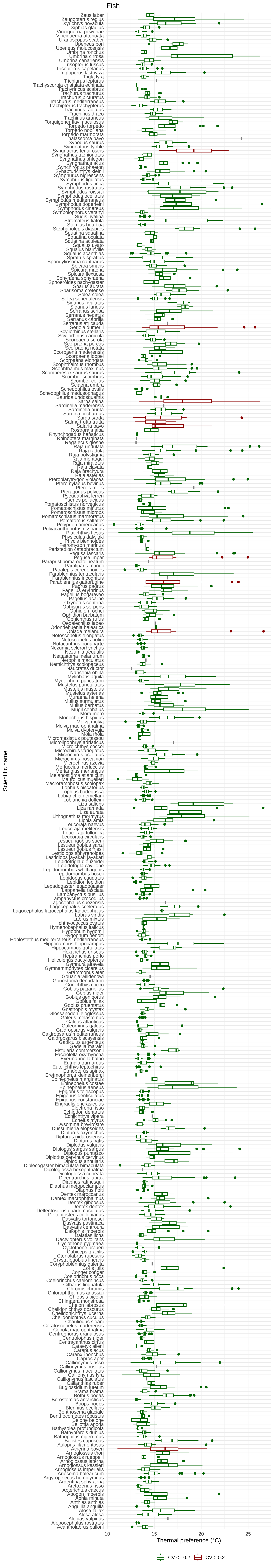
Fish
Scientific name
Thermal preference (°C)
10
15
20
25
CV <= 0.2
CV > 0.2

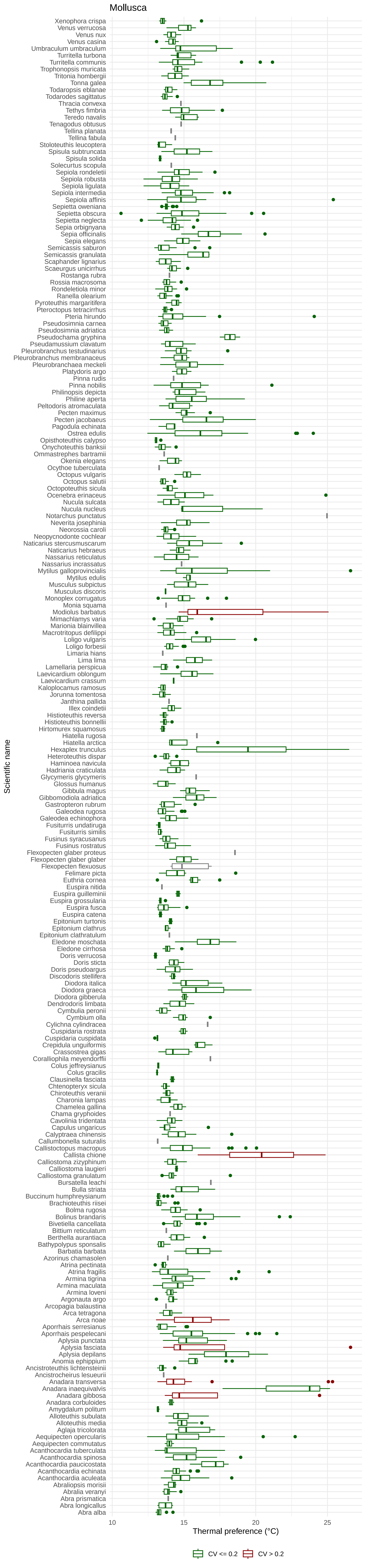
Mollusca
Xenophora crispa
Venus verrucosa
Venus nux
Venus casina
Umbraculum umbraculum
Turritella turbona
Turritella communis
Trophonopsis muricata
Tritonia hombergii
Tonna galea
Todaropsis eblanae
Todarodes sagittatus
Thracia convexa
Tethys fimbria
Teredo navalis
Tenagodus obtusus
Tellina planata
Tellina fabula
Stoloteuthis leucoptera
Spisula subtruncata
Spisula solida
Solecurtus scopula
Sepiola rondeletii
Sepiola robusta
Sepiola ligulata
Sepiola intermedia
Sepiola affinis
Sepietta oweniana
Sepietta obscura
Sepietta neglecta
Sepia orbignyana
Sepia officinalis
Sepia elegans
Semicassis saburon
Semicassis granulata
Scaphander lignarius
Scaeurgus unicirrhus
Rostanga rubra
Rossia macrosoma
Rondeletiola minor
Ranella olearium
Pyroteuthis margaritifera
Pteroctopus tetracirrhus
Pteria hirundo
Pseudosimnia carnea
Pseudosimnia adriatica
Pseudochama gryphina
Pseudamussium clavatum
Pleurobranchus testudinarius
Pleurobranchus membranaceus
Pleurobranchaea meckeli
Platydoris argo
Pinna rudis
Pinna nobilis
Philinopsis depicta
Philine aperta
Peltodoris atromaculata
Pecten maximus
Pecten jacobaeus
Pagodula echinata
Ostrea edulis
Opisthoteuthis calypso
Onychoteuthis banksii
Ommastrephes bartramii
Okenia elegans
Ocythoe tuberculata
Octopus vulgaris
Octopus salutii
Octopoteuthis sicula
Ocenebra erinaceus
Nucula sulcata
Nucula nucleus
Notarchus punctatus
Neverita josephinia
Neorossia caroli
Neopycnodonte cochlear
Naticarius stercusmuscarum
Naticarius hebraeus
Nassarius reticulatus
Nassarius incrassatus
Mytilus galloprovincialis
Mytilus edulis
Musculus subpictus
Musculus discoris
Monoplex corrugatus
Monia squama
Modiolus barbatus
Mimachlamys varia
Marionia blainvillea
Macrotritopus defilippi
Loligo vulgaris
Loligo forbesii
Limaria hians
Lima lima
Lamellaria perspicua
Laevicardium oblongum
Laevicardium crassum
Kaloplocamus ramosus
Jorunna tomentosa
Janthina pallida
Illex coindetii
Histioteuthis reversa
Histioteuthis bonnellii
Hirtomurex squamosus
Hiatella rugosa
Hiatella arctica
Hexaplex trunculus
Heteroteuthis dispar
Haminoea navicula
Hadriania craticulata
Glycymeris glycymeris
Glossus humanus
Gibbula magus
Gibbomodiola adriatica
Gastropteron rubrum
Galeodea rugosa
Galeodea echinophora
Fusiturris undatiruga
Fusiturris similis
Fusinus syracusanus
Fusinus rostratus
Flexopecten glaber proteus
Flexopecten glaber glaber
Flexopecten flexuosus
Felimare picta
Euthria cornea
Euspira nitida
Euspira guilleminii
Euspira grossularia
Euspira fusca
Euspira catena
Epitonium turtonis
Epitonium clathrus
Epitonium clathratulum
Eledone moschata
Eledone cirrhosa
Doris verrucosa
Doris sticta
Doris pseudoargus
Discodoris stellifera
Diodora italica
Diodora graeca
Diodora gibberula
Dendrodoris limbata
Cymbulia peronii
Cymbium olla
Cylichna cylindracea
Cuspidaria rostrata
Cuspidaria cuspidata
Crepidula unguiformis
Crassostrea gigas
Coralliophila meyendorffii
Colus jeffreysianus
Colus gracilis
Clausinella fasciata
Chtenopteryx sicula
Chiroteuthis veranii
Charonia lampas
Chamelea gallina
Chama gryphoides
Cavolinia tridentata
Capulus ungaricus
Calyptraea chinensis
Callumbonella suturalis
Callistoctopus macropus
Callista chione
Calliostoma zizyphinum
Calliostoma laugieri
Calliostoma granulatum
Bursatella leachi
Bulla striata
Buccinum humphreysianum
Brachioteuthis riisei
Bolma rugosa
Bolinus brandaris
Bivetiella cancellata
Bittium reticulatum
Berthella aurantiaca
Bathypolypus sponsalis
Barbatia barbata
Azorinus chamasolen
Atrina pectinata
Atrina fragilis
Armina tigrina
Armina maculata
Armina loveni
Argonauta argo
Arcopagia balaustina
Arca tetragona
Arca noae
Aporrhais serresianus
Aporrhais pespelecani
Aplysia punctata
Aplysia fasciata
Aplysia depilans
Anomia ephippium
Ancistroteuthis lichtensteinii
Ancistrocheirus lesueurii
Anadara transversa
Anadara inaequivalvis
Anadara gibbosa
Anadara corbuloides
Amygdalum politum
Alloteuthis subulata
Alloteuthis media
Aglaja tricolorata
Aequipecten opercularis
Aequipecten commutatus
Acanthocardia tuberculata
Acanthocardia spinosa
Acanthocardia paucicostata
Acanthocardia echinata
Acanthocardia aculeata
Abraliopsis morisii
Abralia veranyi
Abra prismatica
Abra longicallus
Abra alba
Scientific name
10
15
20
25
Thermal preference (°C)
CV <= 0.2
CV > 0.2

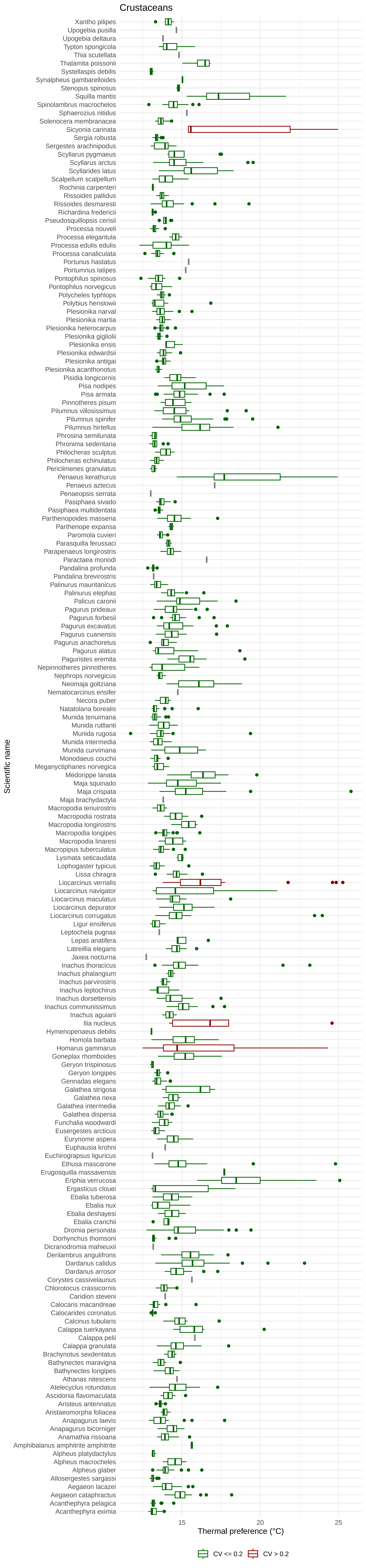
Crustaceans
Xantho pilipes
Upogebia pusilla
Upogebia deltaura
Typton spongicola
Thia scutellata
Thalamita poissonii
Systellaspis debilis
Synalpheus gambarelloides
Stenopus spinosus
Squilla mantis
Spinolambrus macrochelos
Sphaerozius nitidus
Solenocera membranacea
Sicyonia carinata
Sergia robusta
Sergestes arachnipodus
Scyllarus pygmaeus
Scyllarus arctus
Scyllarides latus
Scalpellum scalpellum
Rochinia carpenteri
Rissoides pallidus
Rissoides desmaresti
Richardina fredericii
Pseudosquillopsis cerisii
Processa nouveli
Processa elegantula
Processa edulis edulis
Processa canaliculata
Portunus hastatus
Portumnus latipes
Pontophilus spinosus
Pontophilus norvegicus
Polycheles typhlops
Polybius henslowii
Plesionika narval
Plesionika martia
Plesionika heterocarpus
Plesionika gigliolii
Plesionika ensis
Plesionika edwardsii
Plesionika antigai
Plesionika acanthonotus
Pisidia longicornis
Pisa nodipes
Pisa armata
Pinnotheres pisum
Pilumnus villosissimus
Pilumnus spinifer
Pilumnus hirtellus
Phrosina semilunata
Phronima sedentaria
Philocheras sculptus
Philocheras echinulatus
Periclimenes granulatus
Penaeus kerathurus
Penaeus aztecus
Penaeopsis serrata
Pasiphaea sivado
Pasiphaea multidentata
Parthenopoides massena
Parthenope expansa
Paromola cuvieri
Parasquilla ferussaci
Parapenaeus longirostris
Paractaea monodi
Pandalina profunda
Pandalina brevirostris
Palinurus mauritanicus
Palinurus elephas
Palicus caronii
Pagurus prideaux
Pagurus forbesii
Pagurus excavatus
Pagurus cuanensis
Pagurus anachoretus
Pagurus alatus
Paguristes eremita
Nepinnotheres pinnotheres
Nephrops norvegicus
Neomaja goltziana
Nematocarcinus ensifer
Necora puber
Natatolana borealis
Munida tenuimana
Munida rutllanti
Munida rugosa
Munida intermedia
Munida curvimana
Monodaeus couchii
Meganyctiphanes norvegica
Medorippe lanata
Maja squinado
Maja crispata
Maja brachydactyla
Macropodia tenuirostris
Macropodia rostrata
Macropodia longirostris
Macropodia longipes
Macropodia linaresi
Macropipus tuberculatus
Lysmata seticaudata
Lophogaster typicus
Lissa chiragra
Liocarcinus vernalis
Liocarcinus navigator
Liocarcinus maculatus
Liocarcinus depurator
Liocarcinus corrugatus
Ligur ensiferus
Leptochela pugnax
Lepas anatifera
Latreillia elegans
Jaxea nocturna
Inachus thoracicus
Inachus phalangium
Inachus parvirostris
Inachus leptochirus
Inachus dorsettensis
Inachus communissimus
Inachus aguiarii
Ilia nucleus
Hymenopenaeus debilis
Homola barbata
Homarus gammarus
Goneplax rhomboides
Geryon trispinosus
Geryon longipes
Gennadas elegans
Galathea strigosa
Galathea nexa
Galathea intermedia
Galathea dispersa
Funchalia woodwardi
Eusergestes arcticus
Eurynome aspera
Euphausia krohni
Euchirograpsus liguricus
Ethusa mascarone
Erugosquilla massavensis
Eriphia verrucosa
Ergasticus clouei
Ebalia tuberosa
Ebalia nux
Ebalia deshayesi
Ebalia cranchii
Dromia personata
Dorhynchus thomsoni
Dicranodromia mahieuxii
Derilambrus angulifrons
Dardanus calidus
Dardanus arrosor
Corystes cassivelaunus
Chlorotocus crassicornis
Caridion steveni
Calocaris macandreae
Calocarides coronatus
Calcinus tubularis
Calappa tuerkayana
Calappa pelii
Calappa granulata
Brachynotus sexdentatus
Bathynectes maravigna
Bathynectes longipes
Athanas nitescens
Atelecyclus rotundatus
Ascidonia flavomaculata
Aristeus antennatus
Aristaeomorpha foliacea
Anapagurus laevis
Anapagurus bicorniger
Anamathia rissoana
Amphibalanus amphitrite amphitrite
Alpheus platydactylus
Alpheus macrocheles
Alpheus glaber
Allosergestes sargassi
Aegaeon lacazei
Aegaeon cataphractus
Acanthephyra pelagica
Acanthephyra eximia
Scientific name
15
20
25
Thermal preference (°C)
CV <= 0.2
CV > 0.2

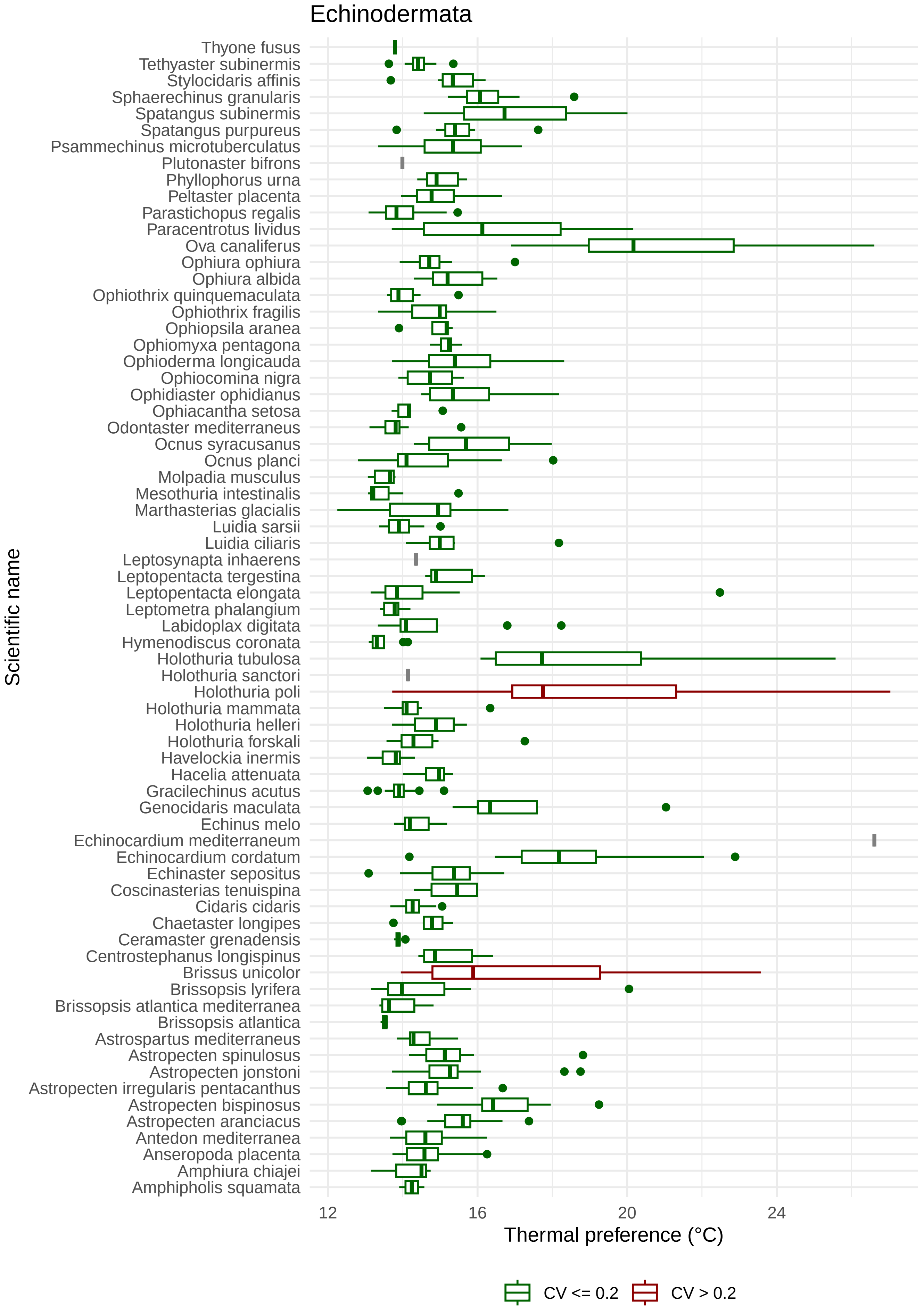

Echinodermata
Thyone fusus
Tethyaster subinermis
Stylocidaris affinis
Sphaerechinus granularis
Spatangus subinermis
Spatangus purpureus
Psammechinus microtuberculatus
Plutonaster bifrons
Phyllophorus urna
Peltaster placenta
Parastichopus regalis
Paracentrotus lividus
Ova canaliferus
Ophiura ophiura
Ophiura albida
Ophiothrix quinquemaculata
Ophiothrix fragilis
Ophiopsila aranea
Ophiomyxa pentagona
Ophioderma longicauda
Ophiocomina nigra
Ophidiaster ophidianus
Ophiacantha setosa
Odontaster mediterraneus
Ocnus syracusanus
Ocnus planci
Molpadia musculus
Mesothuria intestinalis
Marthasterias glacialis
Luidia sarsii
Luidia ciliaris
Leptosynapta inhaerens
Leptopentacta tergestina
Leptopentacta elongata
Leptometra phalangium
Labidoplax digitata
Hymenodiscus coronata
Holothuria tubulosa
Holothuria sanctori
Holothuria poli
Holothuria mammata
Holothuria helleri
Holothuria forskali
Havelockia inermis
Hacelia attenuata
Gracilechinus acutus
Genocidaris maculata
Echinus melo
Echinocardium mediterraneum
Echinocardium cordatum
Echinaster sepositus
Coscinasterias tenuispina
Cidaris cidaris
Chaetaster longipes
Ceramaster grenadensis
Centrostephanus longispinus
Brissus unicolor
Brissopsis lyrifera
Brissopsis atlantica mediterranea
Brissopsis atlantica
Astrospartus mediterraneus
Astropecten spinulosus
Astropecten jonstoni
Astropecten irregularis pentacanthus
Astropecten bispinosus
Astropecten aranciacus
Antedon mediterranea
Anseropoda placenta
Amphiura chiajei
Amphipholis squamata
Scientific name
12
16
20
24
Thermal preference (°C)
CV <= 0.2
CV > 0.2

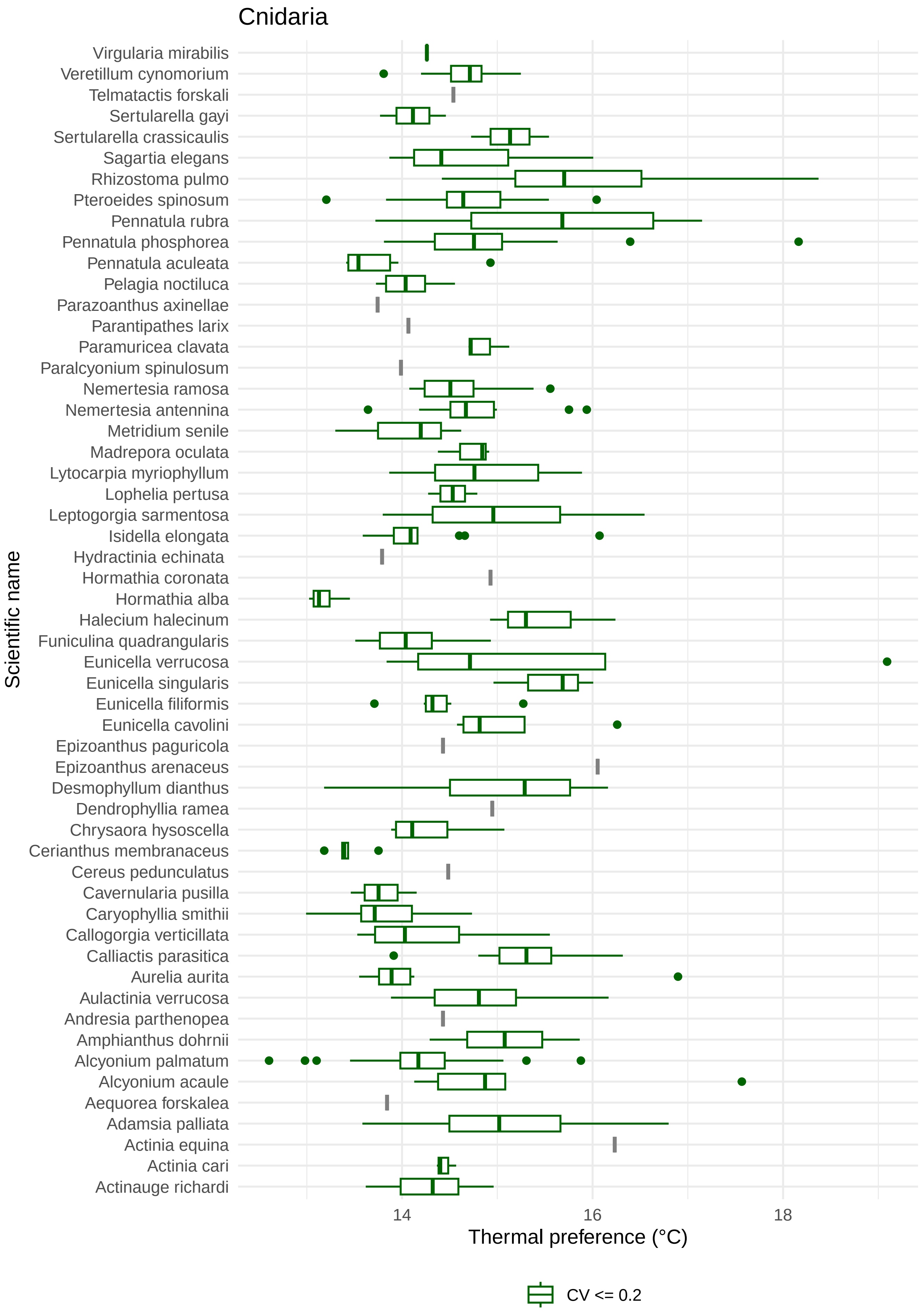

Cnidaria
Virgularia mirabilis
Veretillum cynomorium
Telmatactis forskali
Sertularella gayi
Sertularella crassicaulis
Sagartia elegans
Rhizostoma pulmo
Pteroeides spinosum
Pennatula rubra
Pennatula phosphorea
Pennatula aculeata
Pelagia noctiluca
Parazoanthus axinellae
Parantipathes larix
Paramuricea clavata
Paralcyonium spinulosum
Nemertesia ramosa
Nemertesia antennina
Metridium senile
Madrepora oculata
Lytocarpia myriophyllum
Lophelia pertusa
Leptogorgia sarmentosa
Isidella elongata
Hydractinia echinata
Hormathia coronata
Hormathia alba
Halecium halecinum
Funiculina quadrangularis
Eunicella verrucosa
Eunicella singularis
Eunicella filiformis
Eunicella cavolini
Epizoanthus paguricola
Epizoanthus arenaceus
Desmophyllum dianthus
Dendrophyllia ramea
Chrysaora hysoscella
Cerianthus membranaceus
Cereus pedunculatus
Cavernularia pusilla
Caryophyllia smithii
Callogorgia verticillata
Calliactis parasitica
Aurelia aurita
Aulactinia verrucosa
Andresia parthenopea
Amphianthus dohrnii
Alcyonium palmatum
Alcyonium acaule
Aequorea forskalea
Adamsia palliata
Actinia equina
Actinia cari
Actinauge richardi
Scientific name
14
16
18
Thermal preference (°C)
CV <= 0.2

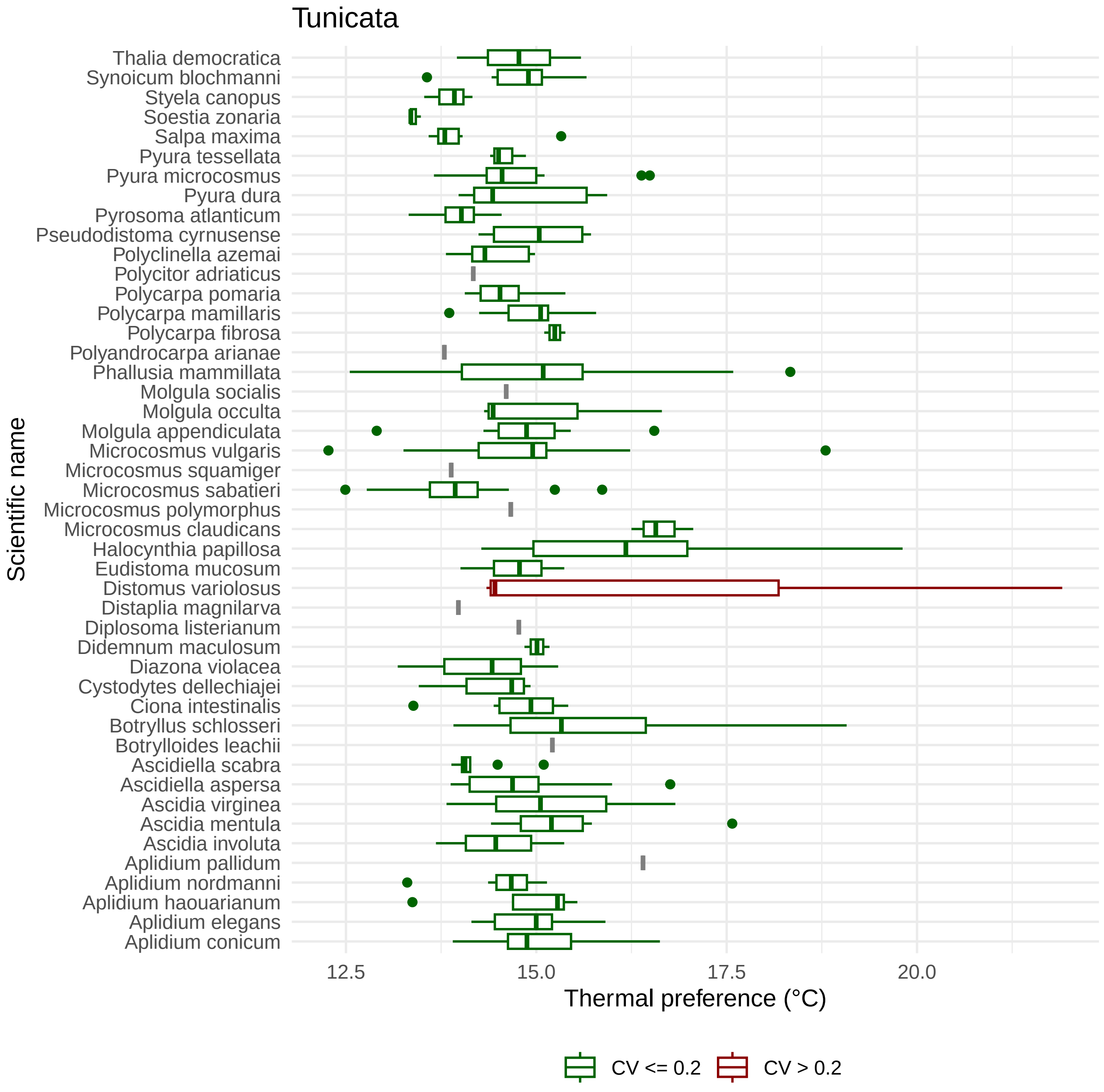
Tunicata
Scientific name
Thalia democratica
Synoicum blochmanni
Styela canopus
Soestia zonaria
Salpa maxima
Pyura tessellata
Pyura microcosmus
Pyura dura
Pyrosoma atlanticum
Pseudodistoma cyrnusense
Polyclinella azemai
Polycitor adriaticus
Polycarpa pomaria
Polycarpa mamillaris
Polycarpa fibrosa
Polyandrocarpa arianae
Phallusia mammillata
Molgula socialis
Molgula occulta
Molgula appendiculata
Microcosmus vulgaris
Microcosmus squamiger
Microcosmus sabatieri
Microcosmus polymorphus
Microcosmus claudicans
Halocynthia papillosa
Eudistoma mucosum
Distomus variolosus
Distaplia magnilarva
Diplosoma listerianum
Didemnum maculosum
Diazona violacea
Cystodytes dellechiajei
Ciona intestinalis
Botryllus schlosseri
Botrylloides leachii
Ascidiella scabra
Ascidiella aspersa
Ascidia virginea
Ascidia mentula
Ascidia involuta
Aplidium pallidum
Aplidium nordmanni
Aplidium haouarianum
Aplidium elegans
Aplidium conicum
12.5
15.0
17.5
20.0
Thermal preference (°C)
CV <= 0.2
CV > 0.2
